# Determination and Kinetic Characterization of a New Potential Inhibitor for AmsI Protein Tyrosine Phosphatase from the Apple Pathogen *Erwinia amylovora*

**DOI:** 10.3390/molecules28237774

**Published:** 2023-11-25

**Authors:** Simone Albani, Ivan Polsinelli, Luca Mazzei, Francesco Musiani, Stefano Benini

**Affiliations:** 1 Laboratory of Bioinorganic Chemistry, Department of Pharmacy and Biotechnology, University of Bologna, 40126 Bologna, Italy; s.albani@fz-juelich.de (S.A.); luca.mazzei2@unibo.it (L.M.); 2 Bioorganic Chemistry and Bio-Crystallography Laboratory (B2Cl), Faculty of Agricultural, Environmental and Food Sciences, Free University of Bolzano, 39100 Bolzano, Italy; ivan.polsinelli@grupposandonato.it; 3 Institute of Molecular and Translational Cardiology (IMTC), IRCCS Policlinico San Donato, 20097 San Donato Milanese, Italy

**Keywords:** *Erwinia amylovora*, fire blight, amylovoran, exopolysaccharide, EPS production regulation, molecular docking, in vitro assays, virtual screening, protein tyrosine phosphatase, inhibition constant

## Abstract

*Erwinia amylovora* is a Gram-negative bacterium, responsible for the fire blight disease in Rosaceae plants. Its virulence is correlated with the production of an exopolysaccharide (EPS) called amylovoran, which protects the bacterium from the surrounding environment and helps its diffusion inside the host. Amylovoran biosynthesis relies on the expression of twelve genes clustered in the *ams* operon. One of these genes, *amsI*, encodes for a Low Molecular Weight Protein Tyrosine Phosphatase (LMW-PTP) called *Ea*AmsI, which plays a key role in the regulation of the EPS production pathway. For this reason, *Ea*AmsI was chosen in this work as a target for the development of new antibacterial agents against *E. amylovora*. To achieve this aim, a set of programs (DOCK6, OpenEye FRED) was selected to perform a virtual screening using a database of ca. 700 molecules. The six best-scoring compounds identified were tested in in vitro assays. A complete inhibition kinetic characterization carried out on the most promising molecule (n-Heptyl β-D-glucopyranoside, N7G) showed an inhibition constant of 7.8 ± 0.6 µM. This study represents an initial step towards the development of new *Ea*AmsI inhibitors able to act as antibacterial agents against *E. amylovora* infections.

## 1. Introduction

Fire blight is a disease affecting plants of the Rosaceae family. Its etiologic agent is the Gram-negative bacterium *Erwinia amylovora*, which is considered one of the ten most dangerous plant pathogens [1]. Among the host plants of this disease, some are of high economic interest, such as pears and apples [2]. Nowadays, fire blight is spreading worldwide, being reported in more than 50 countries [3,4,5,6,7]. Infected plants require pruning and/or eradication, which in some cases results in the complete destruction of orchards [8]. Within the *E. amylovora* genome, the *ams* operon is necessary for pathogenicity and is responsible for the biosynthesis of amylovoran [9], which is a complex branched heteropolysaccharide essential for virulence and pathogenicity. Amylovoran is the major component of the exopolysaccharide (EPS) capsule of the bacterium together with levan [10,11,12,13,14,15,16].

A strain of *E. amylovora* deficient in amylovoran production loses the ability to infect apple plants. Indeed, amylovoran biosynthesis is fundamental for the host-specific pathogenicity of *E. amylovora* [17] and, as such, it is finely regulated. One regulatory pathway involves a Low-Molecular Weight Protein Tyrosine Phosphatase (LMW-PTP) called AmsI (*Ea*AmsI), encoded in the *ams* operon [9]. A balanced regulation of its expression is fundamental for the pathogenesis of the bacterium: *Ea*AmsI overproduction hinders EPS production through the inhibition of EPS biosynthesis, while deletion of the *amsI* gene results in an EPS-deficient bacterium [9]. The central role of *Ea*AmsI in EPS synthesis regulation makes this protein an ideal target to control, in a new and effective way, the spreading of fire blight outbreaks in the field.

**Scheme 1:**
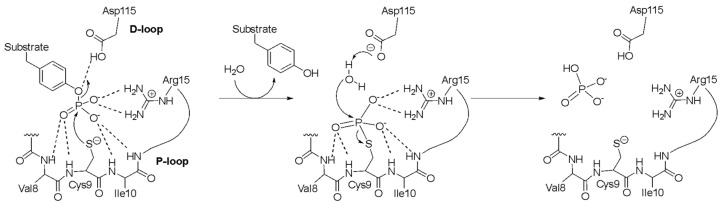
Proposed reaction mechanism of Protein Tyrosine Phosphatases (PTPs). Residues are labeled according to *Ea*AmsI numbering [19,22].

LMW-PTPs are proteins with a molecular weight of about 18 kDa [18] that catalyze the removal of a phosphate group from phosphorylated tyrosine residues of a cognate protein [19]. In general, members of the PTP superfamily have extremely limited sequence similarity, while maintaining a conserved catalytic site [20]. In LMW-PTPs, the catalytic site is characterized by the typical CX5R(S/T) motif [20]; it is found in the so-called P-loop, located at the N-terminus of the protein, where Cys9 and Arg15 (*Ea*AmsI numbering) play an essential role in the catalytic mechanism, together with Asp115, the latter located in the so-called D-loop at the C-terminal portion of the protein [19]. According to the generally accepted LMW-PTP reaction mechanism (Scheme 1), Asp115 acts as a general acid by protonating the phosphoester O atom of the substrate phospho-tyrosine, while the side chain of Cys9, in its thiolate (S^–^) form, is responsible for the nucleophilic attack on the P atom of the substrate and the formation of a phospho-cysteine intermediate, with the release of the dephosphorylated tyrosine. In the second step of the reaction mechanism, Asp115 acts as a general base by activating a water molecule, which subsequently carries out a second nucleophilic attack on the P atom of the phospho-cysteine, thus causing its hydrolysis and the subsequent release of the phosphate group [21].

**Scheme 2:**
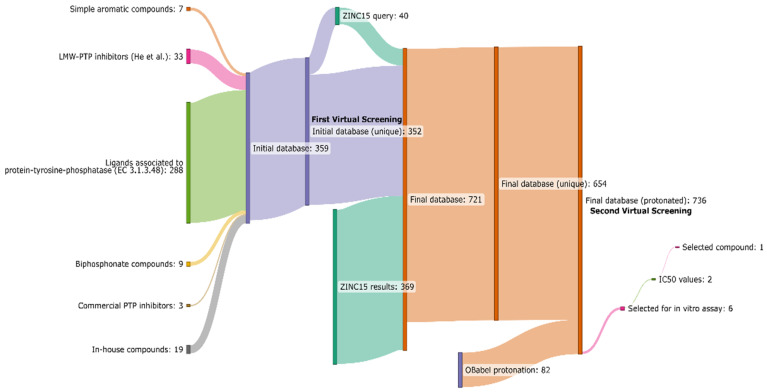
Workflow used in this work for the identification of new putative *Ea*AmsI inhibitors.

In this work, modern computational techniques based on virtual screening procedures were applied for the identification of new compounds able to interact with the active site of *Ea*AmsI, for which a high-resolution X-ray crystal structure is available (PDB id: 4D74) [19]. Scheme 2 offers a summary of the workflow used, which is outlined in more detail in the following sections. In brief, out of ca. 700 candidates, six molecules were selected to be tested in vitro as *Ea*AmsI inhibitors (Scheme 2) and the most promising one, namely n-Heptyl β-D-glucopyranoside (N7G), was then kinetically characterized for its inhibition properties towards the enzyme, revealing a competitive inhibition mechanism with an inhibition constant of ca. 8 µM (see Section 2 below).

With this work, we found a new inhibitor of the LMW-PTP AmsI from the rosaceous phytopathogen *E. amylovora*, thus paving the way for the development of novel *Ea*AmsI inhibitors based on the N7G scaffold with structure-based design and structure–activity relationship studies, with potential fallouts for agronomic purposes.

## 2. Results and Discussion

To select the best software for the Virtual Ligand Screening (VLS), four different freely available docking programs (AutoDock [23], AutoDock Vina [24], OpenEye Fred [25,26], and DOCK6 [27]) were tested in their ability to replicate the ligand pose observed in X-ray crystal structures of three LMW-PTPs with high structural similarity to *Ea*AmsI: LMWPTP-1 from *Vibrio cholerae* bound to 3-(*N-*morpholino)propanesulfonic acid (MOPS) (PDB id: 4LRQ) [28], LMPTP from *Homo sapiens* bound to 2-(*N*-morpholino)ethanesulfonic acid (MES) (PDB id: 5JNT) [29], and LMPTP from *Bos taurus* bound to 4-(2-hydroxyethyl)-1-piperazineethanesulfonic acid (HEPES) (PDB id: 5JNV) [29]. The structures were selected because of the high resolution and good structural alignment with *Ea*AmsI. For each structure, ligands and solvent molecules were removed from the PDB files (Table S1, Figure S1). Water molecule removal was justified because they were located only in the periphery of the binding pocket and in contact with the bulk solvent. The ligands found in the previous structures have pK_a_ values in the range 6.15–7.55. On the other hand, the pH of the crystallization experiments ranged between 5.0 and 6.5. To handle this possible issue, the ligands were tested in both the protonated and unprotonated state (see structures in Scheme S1 in the Supplementary Information) [19,28,29,30,31,32]. The ability of each program to pose the ligand back into the original site was tested through the calculation of the root-mean-square deviation (RMSD) of the non-hydrogen atoms to the experimental pose (Tables S2–S4 in the Supplementary Information). On average, the RMSD for the re-docking of the experimental ligands was 1.9, 1.5, 0.9, and 0.6 Å for AutoDock, AutoDock Vina, OpenEye Fred, and DOCK6, respectively. In the specific case of *Ea*AmsI, AutoDock, and AutoDock Vina generally failed to correctly reproduce the experimental binding poses. In only a single case (i.e., unprotonated MOPS), AutoDock was able to achieve a binding pose with an RMSD below 1.0 Å. AutoDock Vina performed slightly better, but with very inhomogeneous performance depending on the ligand. On the other hand, OpenEye Fred and DOCK6 were able to reproduce the largest part of the experimental poses with good precision, with an RMSD below 1.0 Å. Thus, OpenEye Fred and DOCK6 were selected as the preferred software for the virtual screening and were further tested with a protonation state of the protein at pH 5.5, 6.5, and 7.4, calculated using the H++ web server (https://biophysics.cs.vt.edu, accessed on 19 October 2019), to assess the effect of an acidic, slightly acidic, and slightly basic pH, respectively (Tables S2 and S4) [33]. The use of a processed protein structure with a specified pH kept the RMSD values in the same order of magnitude, with only slight improvements. The greatest differences were observed for the docking of MES in chain C of *V. cholerae* LMWPTP-1. In this case, there is a large RMSD improvement for DOCK6, but, on the other hand, a worsening for the protonated ligand docked to the protein at pH 5.5.

For the virtual screening, an initial database of 352 molecules was generated from heterogeneous sources. First, known protein-tyrosine-phosphatase (EC 3.1.3.48) ligands were extracted from the PDB by performing a search for known ligands and excluding metal ions, metal-containing anions (such as vanadate or tungstate ions), and ligands with a molecular weight smaller than 76 g mol^−1^. The latter was merged with a list of 33 molecules obtained in a study by He et al. on the inhibition of a human LMW-PTP73 [34]. Finally, an additional group of simple aromatic compounds with a negatively charged moiety was added to the database, due to their chemical similarity with phosphotyrosine. The initial library was docked using DOCK6 and OpenEye FRED on the *Ea*AmsI structure at pH 5.5 (i.e., the pH at which the experimental essays are performed) and screened to obtain a selection of 40 compounds based on the corresponding GRID and Chemgauss scores of the two programs, respectively [25,27]. Compounds in this list were then used as queries in the ZINC15 database to search for analogous structures, thus generating a new set of 369 molecules, used to expand the virtual screening library.

**Figure 1:**
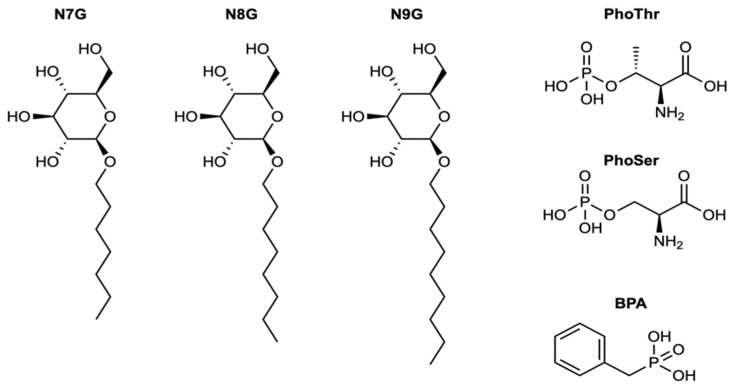
Two-dimensional diagrams and names of the six selected molecules resulting from the virtual screening.

The molecules in the library were protonated at pH 5.5 and compounds were subsequently docked to the *Ea*AmsI structure as it was prepared for the initial virtual screening. For each compound, the best Chemgauss and GRID score was calculated. The virtual screening returned a list containing a total of 80 high-scoring structures. Half of these structures were derived from the first database of in-house compounds and known PTP ligands, while the other half was from the ZINC15 [35] website of commercially available compounds. We selected a group of molecules among the top-ranking compounds for further evaluation and conducted preliminary tests alongside commercially available PTP inhibitors (PTP inhibitor IV, V, and XVIII obtained from *Calbiochem*), which served as benchmarks. In the end, six commercially available compounds ready for purchase were selected for further in vitro studies (Figure 1 and Table 1).

**Figure 2:**
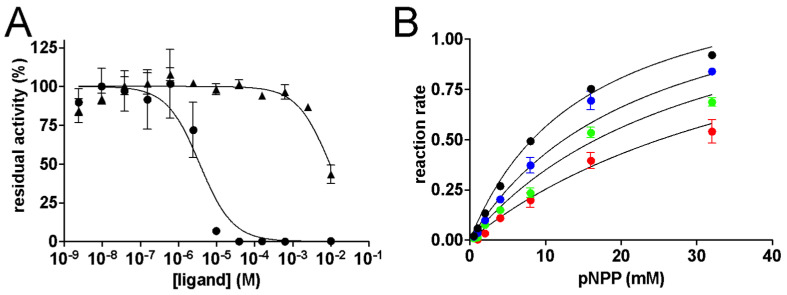
(**A**) Dose–response curves of *Ea*AmsI activity in the presence of N7G (black circles) and N8G (black triangles). (**B**) Michaelis–Menten plot of *Ea*AmsI at increasing concentrations of N7G (0, 4, 8, and 16 µM represented as black, blue, green, and red dots, respectively). The lines represent the results of the data-fitting procedure described in the experimental section.

The ability of the six compounds identified by virtual screening to inhibit *Ea*AmsI was preliminarily assessed by the estimation of IC_50_ values. Among the tested molecules, N7G and N8G showed detectable inhibition, with IC_50_ values of 3.6 ± 0.8 μM and 9.5 ± 1.3 mM, respectively (Figure 2A). In particular, the IC_50_ value of N7G is comparable to those of other commercially available PTP inhibitors (PTP IV, V, XVIII, *Calbiochem*) [29,30,31] so a full kinetic characterization of its inhibition was carried out, while N8G was not further investigated due to its relatively low inhibitory effect.

To kinetically characterize the inhibition of N7G on *Ea*AmsI, the enzymatic rates were measured as a function of substrate concentration in the absence and in the presence of increasing concentrations of the inhibitor. The resulting experimental data, successfully fitted using the competitive inhibition model of the Michaelis–Menten equation (Figure 2B), resulted in the following kinetic parameters: *k*_cat_ = 111 ± 3 s^−1^, *K*_M_ = 15.1 ± 1.3 mM, K_I_ = 7.8 ± 0.6 µM. Both the estimated *k*_cat_ and *K*_M_ values differ from those previously reported (34.4 s^−1^ and 1.3 mM in citrate buffer at pH 5.5, respectively) [19]. These differences can be ascribed to the presence of 200 mM NaCl in the reaction mixture, as the ionic strength dependence of *k*_cat_ and *K*_M_ for the PTP from *Yersina enterocolitica* was reported to range from ca. 500 to 1104 s^−1^ and from ca. 1 to 58 mM, respectively, by increasing the ionic strength from 20 mM to 1.5 M [29]. Another possible element that could have shifted the kinetic parameters is the Cl^−^/pNPP competition, as chloride ions have been previously reported in the phosphate-binding site of the active-site region in other bacterial PTPs, as in *Mycobacterium tuberculosis* (PDB ids: 1U2P and 1U2Q [36]) and *Streptococcus pyogenes* (PDB id: 5GOT [37]).

**Figure 3:**
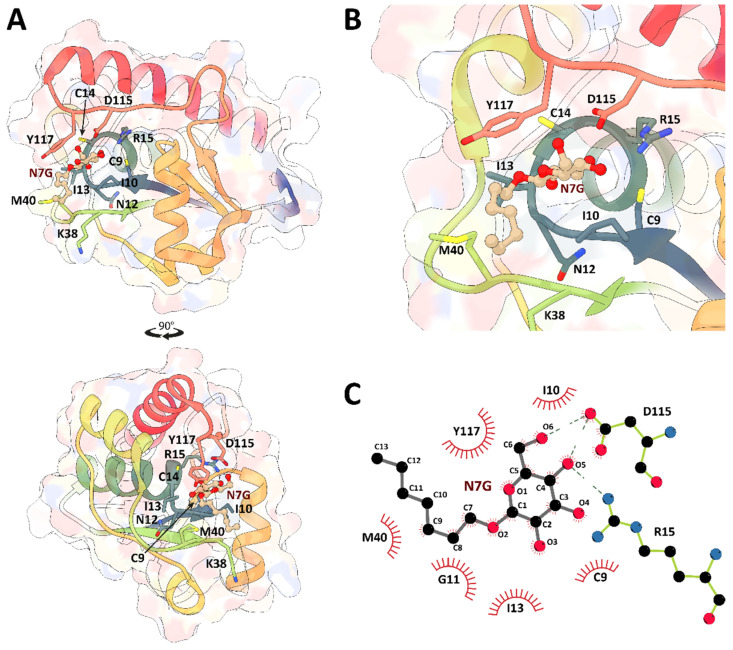
(**A**) Cartoon and molecular surface representation of *Ea*AmsI bound to N7G as a result of the DOCK6 molecular docking. *Ea*AmsI cartoons are colored from blue to red going from the N- to the C-terminal. Residues involved in the reaction or in the binding of N7G are sticks, while N7G is in ball-and-stick (carbon, light orange; oxygen, red). (**B**) Detail of N7G binding pose in the *Ea*AmsI binding site. (**C**) Scheme of the interactions between the docked N7G molecule and *Ea*AmsI. The scheme represents H-bonds (dashed lines) and van der Waals interactions (red spiked arcs).

The docking procedure predicted a favorable binding of N7G to the *Ea*AmsI binding pocket. This interaction is mediated by the glucose portion of N7G, with O6 and O5 atoms interacting specifically with the catalytically relevant residues Asp115 and Arg15 (Figure 3). The binding poses of the other compounds short-listed from the virtual screening are summarized in Figure S2.

In conclusion, the kinetic characterization demonstrated a competitive inhibition mechanism that fully validated the results obtained by the computational work and provided quantitative evidence of the ligand’s strong affinity for binding to the free enzyme at the catalytic site.

## 3. Materials and Methods

### 3.1. Selection of the Docking Software

Three homologous structures were selected from the PDB databank based on the jFATcat rigid score [38] of the alignment to *Ea*AmsI. Ligands were redocked to the corresponding enzyme using four different programs, namely OpenEye Fred [25,26], DOCK6 (v. 6.7) [27], Autodock (v. 4.2.6.) [23], and Autodock Vina (v. 1.1.2) [24]. Two different sets of grid spacing values were tested for AutoDock (0.375 and 0.037 Å) and DOCK6 (0.1 and 0.05 Å).

### 3.2. OpenEye Fred Docking

Ligand conformers were generated using OpenEye OMEGA (http://www.eyesopen.com, accessed on 18 October 2023). Conformers with internal clashes and duplicates were discarded by the software, and the remaining ones were clustered based on the root mean square deviation (RMSD). For this virtual screening, a maximum of 200 conformers per compound, clustered with an RMSD of 0.5 Å, was used. Each conformer is docked by OpenEye FRED in the negative image of the active site of the target protein. This negative image was prepared from the structure with PDB id 4D74, using the apopdb2receptor tool in the OpenEye docking suite v3.2 [25,26].

### 3.3. DOCK6 Docking

The database of molecules was protonated to a pH of 5.5 using OpenBabel. The “Anchor and Grow” algorithm in DOCK6 [27] was used, with the following parameters: max_orientations: 1000, min_anchor_size: 5, pruning_use_clustering: yes, pruning_max_orients: 1000, pruning_clustering_cutoff: 100, pruning_conformer_score_cutoff: 100. The parameters for the creation of the grid were compute_grids: yes, grid_spacing 0.05, energy_cutoff_distance: 9999, atom_model: a, attractive_exponent: 6, repulsive_exponent: 12, distance_dielectric: yes, dielectric_factor: 4, bump_filter: yes, bump_overlap: 0.75. In this case, the *Ea*AmsI structure was obtained from the PDB entry with id 4D74. The cubic box was centered to the catalytic cysteine and the side length was set to 14 Å.

### 3.4. Autodock

Polar hydrogen atoms and Kollman charges were added and assigned to the receptor, respectively, using UCSF Chimera 17.1 [39]. A total of 400 grid points, with a variable length of spacing (0.375 and 0.037 Å), were set in each dimension. The center of the crystallized ligand was set as the center of the grid box and the box side was set to 10.0 Å. The AutoGrid4.2 default parameters were used in AutoGrid4 to calculate the affinity map of each atom, the desolvation map, and the electrostatic map. Lamarckian Genetic Algorithm (LGA) was used to search the conformations. Genetic algorithm (GA) was run for 10 sets and the maximum number of energy evaluations was set to 2,500,000 in each run. The maximum number of generations simulated during each GA run was set to 27,000. The rates of the gene mutation and crossover were set to 0.02 and 0.8, respectively. The mean Cauchy distribution for gene mutation was set to 0. The variance of Cauchy distribution for gene mutation was set to 1. The external grid energy was set to 1000. The maximum allowable initial energy was 0. The maximum number of retries was 10,000.

### 3.5. AutoDock Vina

The box was defined as in the AutoDock case. The exhaustiveness of the global search was set to 8. The maximum number of binding modes was 9. The maximum energy difference between the best binding mode and the worst one displayed was set to 3 kcal mol^−1^. The scoring function weights and terms were set as default.

### 3.6. Production of Recombinant EaAmsI

*Ea*AmsI was expressed following the protocol already described in Benini et al. [40]. In brief, the gene was amplified using PCR from *E. amylovora* strain Ea273 (ATCC 49946), cloned into a pETM-11 expression vector [41], and transformed into *Escherichia coli* BL21 (DE3) for protein expression. The purification strategy consisted of an immobilized metal-affinity (IMAC) chromatographic step conducted using a HisTrap HP 5 mL column (GE Healthcare, Uppsala, Sweden) previously equilibrated with 50 mM Tris-HCl, 300 mM NaCl, 20 mM imidazole, 1 mM TCEP, and 5% glycerol at pH 8.0. The enzyme was eluted with 50 mM Tris-HCl, 300 mM NaCl, 150 mM imidazole, 1 mM TCEP, and 5% glycerol at pH 8.0, and further purified by size-exclusion chromatography using a Superdex 75 16/600 GL column (GE Healthcare, Sweden) equilibrated with 50 mM Tris–HCl, 300 mM NaCl, 5% glycerol, and 1 mM TCEP at pH 7.8. Fractions of clean and monodisperse *Ea*AmsI were pooled together, concentrated up to 2 mg mL^−1^, and stored at −80 °C for further studies.

### 3.7. Kinetic Measurements

All the kinetic measurements reported here were conducted at room temperature and carried out in triplicates.

IC_50_ assays were carried out in 200 μL reaction mixtures containing 200 ng mL^−1^ *Ea*AmsI and increasing concentrations (in the range 0.1 nM–10 mM) of each compound listed in Table 1, dissolved in 100 mM citrate buffer, at pH 5.5. After ten minutes of pre-incubation, the enzymatic reaction was started by adding 4 mM p-nitrophenyl phosphate (pNPP, purchased from Sigma–Aldrich) as a substrate. Enzymatic activity was measured through a spectrophotometric assay, performed using a Tecan Infinite 200 PRO microplate reader and 96-well microplates, by following the overtime change in absorbance at 405 nm due to the formation of p-nitrophenol (pNP, molar extinction coefficient at 405 nm = 18,000 M^−1^cm^−1^) from the hydrolysis of pNPP. The reaction proceeded for 15 min prior to quenching with 50 μL of 1M NaOH. The initial reaction rates (*v_i_*) for each set of samples were calculated as the slopes of the linear portions of the absorbance vs. time curves, and the resulting averaged value was normalized with respect to the average initial reaction rate in the absence of any compound (*v*_0_). The obtained percentage residual activity values were plotted as a function of the inhibitor concentration on a semi-log graph, and the inhibitor concentration values leading to a 50% inactivation of the enzyme (IC_50_) were then estimated by fitting the resulting data with the canonical dose–response curve for the calculation of the IC_50_ values available in the Prism v. 8.4.3 software (https://www.graphpad.com/, accessed on 18 October 2023).

Michaelis–Menten kinetics describing *Ea*AmsI activity as a function of substrate concentration in the absence or in the presence of increasing concentrations of N7G (4, 8, 16 uM) were carried out in 200 μL reaction mixtures containing 200 ng mL^−1^ *Ea*AmsI in 100 mM citrate buffer and 200 mM NaCl at pH 5.5. Each enzymatic reaction was started by the addition of pNPP used in a concentration range of 0.5–64 mM and proceeded for 5 min prior to quenching with 50 μL of 1 M NaOH. The pNP production over time was quantified using a path length of 0.65 cm, corresponding to 250 µL of solution in a well of 3.48 mm radius (following manufacturer data sheet, VWR International). The averaged initial reaction rate measured at each substrate concentration (*v_i_*) was plotted as a function of substrate concentration, and data analysis was performed by simultaneously fitting all the available data using the competitive inhibition model of the Michaelis–Menten curve available in the Prism v. 8.4.3 software, obtaining shared values for *k*_cat_, *K*_M_, and *K*_I_.

### 3.8. Software for Schemes and Analyses

Scheme 1 and molecular schemes were made using ChemDraw 22.0.0 (https://revvitysignals.com/products/research/chemdraw, accessed on 18 October 2023). Scheme 2 was prepared by using SankeyMATIC (https://sankeymatic.com/, accessed on 18 October 2023). UCSF Chimera 17.1 was used for the rendering of EaAmsI bound to N7G, while 2D interaction diagrams were prepared using LigPlot+ v.2.2 [42].

## 4. Conclusions

In this work, an integrated computational and biochemical study was carried out with the aim of identifying new molecules potentially acting as inhibitors of AmsI from *E. amylovora*, an enzyme involved in the regulation of the amylovoran synthesis pathway and with a key role in bacterial pathogenesis. A workflow involving a ligand virtual screening and biochemical assays led to the identification of N7G, a ligand able to inhibit *Ea*AmsI with a competitive inhibition mechanism and a *K*_I_ in the low micromolar range. The results reported in this work indicate that N7G has great potential as a lead compound toward the discovery of novel chemical treatments against this plant pathogen. Moreover, this work provides a significant step towards the development of alternative strategies against *E. amylovora* infections which do not rely on conventional antibiotics, the latter being forbidden in many countries because of antibiotic resistance issues. Additional research, currently ongoing in our laboratory, will be beneficial for full comprehension of the effects of N7G binding in the active site of *Ea*AmsI, including in vivo testing, as well as further investigation of its therapeutic potential.

## Figures and Tables

**Table 1:** Names, IUPAC names, DOCK and Fred docking scores, and LogP estimates of the selected compounds.

Name	IUPAC Name	DOCK6 Score	FRED Score	LogP
BPA	benzylphosphonic acid	−26.72	−10.11	0.55
PhoThr	(2S,3R)-2-amino-3-phosphonooxybutanoic acid	−26.42	−10.97	−1.33
PhoSer	(2S)-2-amino-3-(phosphonooxy)propanoic acid	−27.38	−9.29	−1.75
N7G	(2R,3R,4S,5S,6R)-2-(heptyloxy)-6- (hydroxymethyl)oxane-3,4,5-triol	−33.50	−4.66	0.37
N8G	(2R,3S,4S,5R,6R)-2-(hydroxymethyl)-6- (octyloxy)oxane-3,4,5-triol	−33.22	−4.26	0.81
N9G	(2R,3S,4S,5R,6R)-2-(hydroxymethyl)-6- (nonyloxy)oxane-3,4,5-triol	−32.81	−4.31	1.26

## Data Availability

The complete library in SMILES format, docking scores, and data points for the kinetic assay are available upon request. Interested parties are kindly invited to contact the corresponding authors to request access to the aforementioned data.

## Supplementary Materials

The following supporting information can be downloaded at: https://www.mdpi.com/article/10.3390/molecules28237774/s1, Table S1: Selection of structures for redocking evaluation, Figure S1: Alignment of structures selected for redocking [43,44], Scheme S1: Chemical structures of molecules used for redocking, Table S2: OpenEye Fred redocking evaluation, Table S3: Autodock and Autodock Vina redocking evaluation, Table S4: DOCK6 redocking evaluation, Figure S2: Scheme of the interactions between the docked PhoSer, PhoThr, N8G, N9G, and BPA molecules and *Ea*AmsI. An additional table in CSV format reporting all the compounds used in the virtual screening together with the ZINC IDs or the compound IDs for in-house compounds, the SMILE, and the docking scores for DOCK6 and OpenEye FRED is also available as a separate file.
