# Supplementary material for: Determination and Kinetic Characterization of a New Potential Inhibitor for AmsI Protein Tyrosine Phosphatase from the Apple Pathogen Erwinia amylovora
Source: Molecules. 2023 Nov 25;28(23):7774. doi: 10.3390/molecules28237774 (PMC10708540; doi:10.3390/molecules28237774)
Supplement: Supplementary file 1 [file molecules-28-07774-s001.zip › molecules-2699274-supplementary.pdf]

SUPPORTING INFORMATION

Determination and kinetic characterization of a new potential inhibitor for AmsI protein tyrosine phosphatase from the apple pathogen *Erwinia amylovora*

Simone Albani[1,2,+], Ivan Polsinelli[3,+], Luca Mazzei[1], Francesco Musiani[1,*], Stefano Benini[3,*]

[1] Laboratory of Bioinorganic Chemistry, Department of Pharmacy and Biotechnology, University of Bologna, Italy
[2] Present address: Institute for Neuroscience and Medicine (INM-9), Forschungszentrum Jülich, Jülich, 52425, Germany
[3] Bioorganic chemistry and Bio-Crystallography laboratory (B$_2$Cl), Faculty of Agricultural, Environmental and Food Sciences, Free University of Bolzano, 39100 Bolzano, Italy
+ These authors equally contributed to this work.
* Correspondence: francesco.musiani@unibo.it; stefano.benini@unibz.it

Table S1. Selection of protein structures for redocking [1–3], jFATCat rigid score [4] and sequence identity (SI) with respect to *Ea*AmsI.

PDB id	Organism	Resolution	Crystal pH	Ligand IUPAC name	jFATCat rigid score	SI (%)
4D74	*E. amylovora*	1.57 Å	5.5	-	432.00	100%
4LRQ	*V. cholerae* O395	1.45 Å	5.5	3[N-morpholino]propane sulfonic acid	348.88	28.38%
5JNT	*Homo Sapiens*	1.45 Å	6.5	2-(N-morpholino)-ethane sulphonic acid	339.63	25.97%
5JNV	*Bos taurus*	1.6 Å	6.5	4-(2-hydroxyethyl)-1-piperazine ethane sulphonic acid	341.00	27.74%

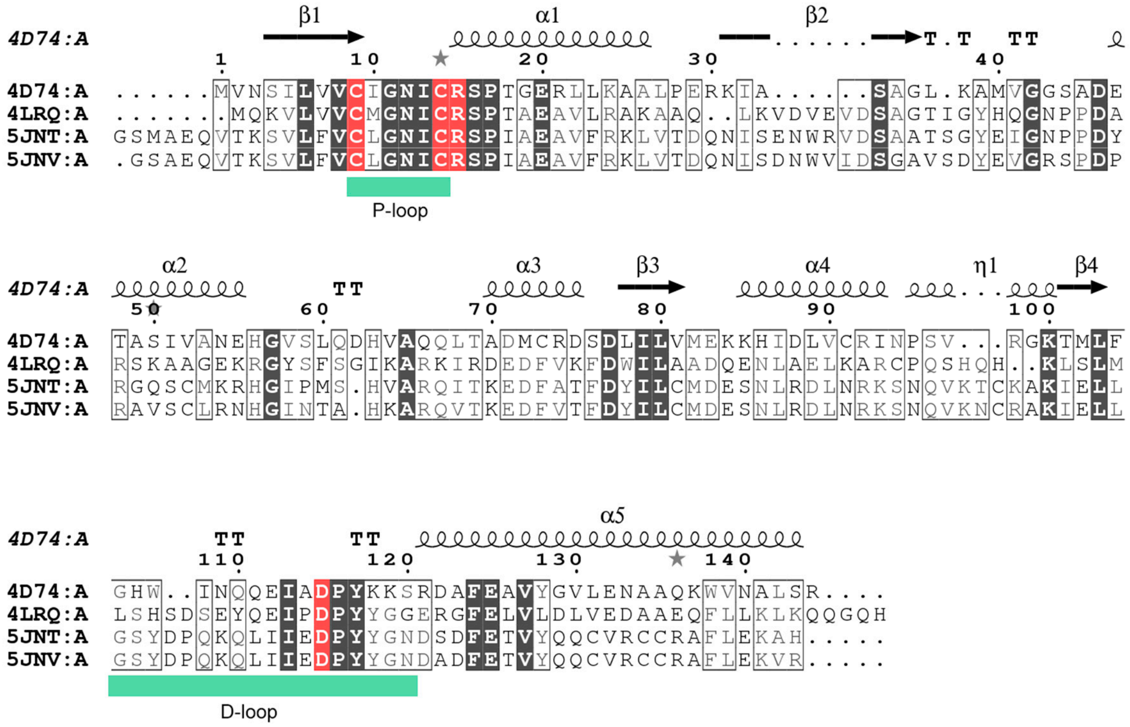

Figure S1. Alignment of selected structures for redocking. The alignment was generated with Clustal Omega[5], and the visualisation with ESPript 3.0[6]. Catalytic residues are highlighted in red, conserved residues in black, and P-loop and D-loop are delimited by green boxes.

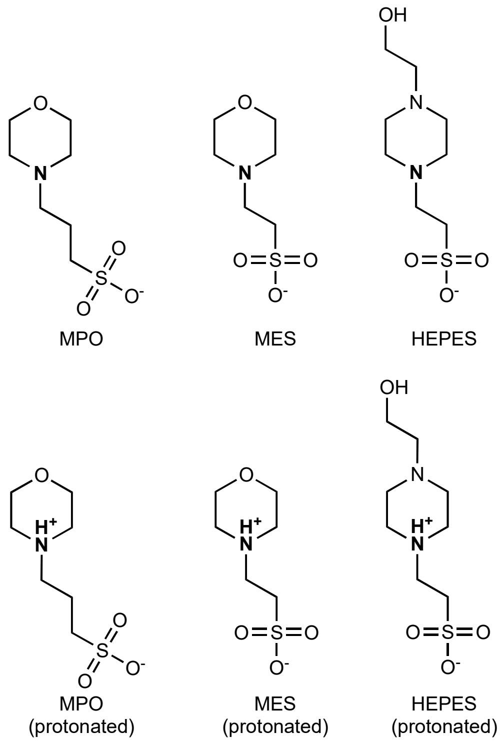

Scheme S1. Ligands used in molecular docking for software evaluation, and corresponding working names, in the two protonation states.

Table S2. RMSD of the poses docked by OpenEye FRED. Different protonation states of the protein structure were simulated using the H++ webserver and represented in each column.

**OpenEye FRED**					
Protein structure pH:		unspecified	5.5	6.5	7.4
Structure	Ligand	RMSD (Å)	RMSD (Å)	RMSD (Å)	RMSD (Å)
4LRQ.c	MPO	0.400	0.519	-	0.519
	MPO (protonated)	0.367	3.150	-	0.495
5JNT	MES	0.400	0.395	0.395	0.390
	MES (protonated)	1.047	1.119	1.119	1.105
5JNV	HEPES	1.098	-	0.943	0.943
	HEPES (protonated)	0.833	-	0.742	0.742

Table S3. RMSD of the poses docked by AutoDock Vina and AutoDock.

		**AutoDock Vina**	**AutoDock**	
Grid Spacing:			0.375 Å	0.037 Å
Structure	Ligand	RMSD (Å)	RMSD (Å)	RMSD (Å)
4LRQ.C	MPO	0.780	0.711	1.835
	MPO (protonated)	0.875	1.781	1.736
5JNT	MES	1.154	1.877	1.72
	MES (protonated)	1.384	2.118	1.872
5JNV	HEPES	2.354	2.447	1.785
	HEPES (protonated)	1.876	2.056	1.390

Table S4. RMSDs of the poses docked by DOCK6.

**DOCK6**					
Protein structure pH:		Unspecified	5.5	6.5	7.4
Grid spacing:		0.1 Å	0.05 Å	0.05 Å	0.05 Å
Structure	Ligand	RMSD (Å)	RMSD (Å)	RMSD (Å)	RMSD (Å)
4LRQ.c	MPO	1.110	0.166	-	0.190
	MPO (protonated)	1.110	0.629	-	0.184
5JNT	MES	0.280	0.270	0.598	0.598
	MES (protonated)	0.262	0.346	0.416	0.416
5JNV	HEPES	0.936	-	0.872	0.872
	HEPES (protonated)	0.656	-	0.570	0.570

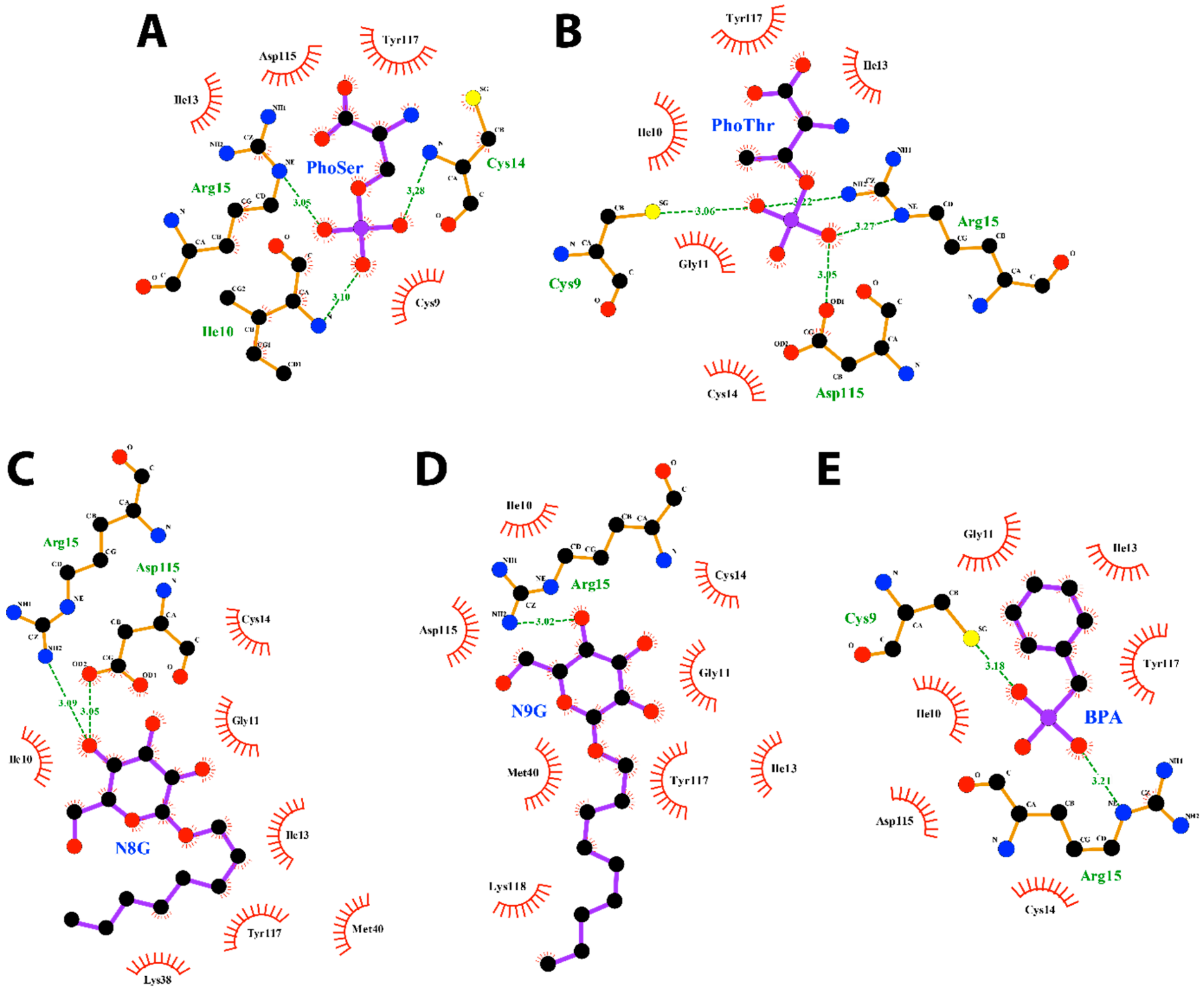

Figure S2. Scheme of the interactions between the docked PhoSer (**A**), PhoThr (**B**), N8G (**C**), N9G (**D**), and BPA (**E**) molecules and *Ea*AmsI. The scheme represents H-bonds (dashed lines) and van der Waals interactions (red spiked arcs).

References

1. Salomone-Stagni, M.; Musiani, F.; Benini, S. Characterization and 1.57 Å Resolution Structure of the Key Fire Blight Phosphatase AmsI from Erwinia Amylovora. *Acta Crystallogr. Sect. F Struct. Biol. Commun.* **2016**, *72*, 903–910, doi:10.1107/S2053230X16018781.
2. Nath, S.; Banerjee, R.; Sen, U. Atomic Resolution Crystal Structure of VcLMWPTP-1 from Vibrio Cholerae O395: Insights into a Novel Mode of Dimerization in the Low Molecular Weight Protein Tyrosine Phosphatase Family. *Biochem. Biophys. Res. Commun.* **2014**, *450*, 390–395, doi:10.1016/j.bbrc.2014.05.129.
3. Stanford, S.M.; Aleshin, A.E.; Zhang, V.; Ardecky, R.J.; Hedrick, M.P.; Zou, J.; Ganji, S.R.; Bliss, M.R.; Yamamoto, F.; Bobkov, A.A.; et al. Diabetes Reversal by Inhibition of the Low-Molecular-Weight Tyrosine Phosphatase. *Nat. Chem. Biol.* **2017**, *13*, 624–632, doi:10.1038/nchembio.2344.
4. Prlić, A.; Bliven, S.; Rose, P.W.; Bluhm, W.F.; Bizon, C.; Godzik, A.; Bourne, P.E. Pre-Calculated Protein Structure Alignments at the RCSB PDB Website. *Bioinformatics* **2010**, *26*, 2983–2985, doi:10.1093/bioinformatics/btq572.
5. Fast, Scalable Generation of High-quality Protein Multiple Sequence Alignments Using Clustal Omega | Molecular Systems Biology Available online: https://www.embopress.org/doi/full/10.1038/msb.2011.75 (accessed on 6 July 2023).
6. Robert, X.; Gouet, P. Deciphering Key Features in Protein Structures with the New ENDscript Server. *Nucleic Acids Res.* **2014**, *42*, W320–W324.
